# Extracellular Vesicles Linking Inflammation, Cancer and Thrombotic Risks

**DOI:** 10.3389/fcell.2022.859863

**Published:** 2022-03-17

**Authors:** Sarah Beck, Bernhard Hochreiter, Johannes A. Schmid

**Affiliations:** ^1^ Institute of Vascular Biology and Thrombosis Research, Center for Physiology and Pharmacology, Medical University of Vienna, Vienna, Austria; ^2^ Institute of Experimental Biomedicine, University Hospital Würzburg and Rudolf Virchow Center for Integrative and Translational Bioimaging, University of Würzburg, Würzburg, Germany

**Keywords:** extracellular vesicles, exosomes, microvesicles, inflammation, cancer, thrombosis

## Abstract

Extracellular vesicles (EVs) being defined as lipid-bilayer encircled particles are released by almost all known mammalian cell types and represent a heterogenous set of cell fragments that are found in the blood circulation and all other known body fluids. The current nomenclature distinguishes mainly three forms: microvesicles, which are formed by budding from the plasma membrane; exosomes, which are released, when endosomes with intraluminal vesicles fuse with the plasma membrane; and apoptotic bodies representing fragments of apoptotic cells. Their importance for a great variety of biological processes became increasingly evident in the last decade when it was discovered that they contribute to intercellular communication by transferring nucleotides and proteins to recipient cells. In this review, we delineate several aspects of their isolation, purification, and analysis; and discuss some pitfalls that have to be considered therein. Further on, we describe various cellular sources of EVs and explain with different examples, how they link cancer and inflammatory conditions with thrombotic processes. In particular, we elaborate on the roles of EVs in cancer-associated thrombosis and COVID-19, representing two important paradigms, where local pathological processes have systemic effects in the whole organism at least in part *via* EVs. Finally, we also discuss possible developments of the field in the future and how EVs might be used as biomarkers for diagnosis, and as vehicles for therapeutics.

## Introduction

The term “extracellular vesicles” designates a highly diverse group of spheres that are surrounded by a lipid bilayer and that are released by a great variety of cells to their respective outsides. Traditionally they were sub-grouped into three main entities: *1*) microvesicles (MVs), also named microparticles or ectosomes, which pinch-off from the plasma membrane and usually have a diameter in the range of 100–1000 nm. *2*) exosomes of 30–150 nm diameter, which are released by fusion of multivesicular bodies (late endosomes with internal vesicles) with the plasma membrane; and *3*) apoptotic bodies released from cells undergoing apoptosis, which are mostly larger than MVs with a diameter in the range of 50–5000 nm. More recently, the various extracellular vesicles released from apoptotic cells have been further subdivided into apoptotic microvesicles and apoptotic exosomes besides the larger apoptotic bodies (Sanwlani and Gangoda, 2021). Since the sizes of these different sub-groups and their general features overlap significantly, it has always been difficult to assign certain biological roles or effects specifically to distinct sub-categories. Furthermore, the originating cell-type as well as the physiological (or pathological) state these cells were in when they released vesicles are crucial. Accordingly, EVs are often more specifically characterized as platelet-derived, endothelial cell-derived and so on, or they are named according to the cellular state (e.g. as “oncosomes” released from cancer cells) or the originating tissue (e.g. “prostasomes” coming from the prostate). Over the years, it became clear that practically all cell types are able to release EVs into all different types of extracellular environment, including blood, urine, semen, saliva, breast milk, etc. Moreover they can exert biological effects not only locally in the microenvironment of releasing cells, but also across larger distances, for instance, when they are distributed *via* the blood circulation (Colombo et al., 2014).

**FIGURE 1:**
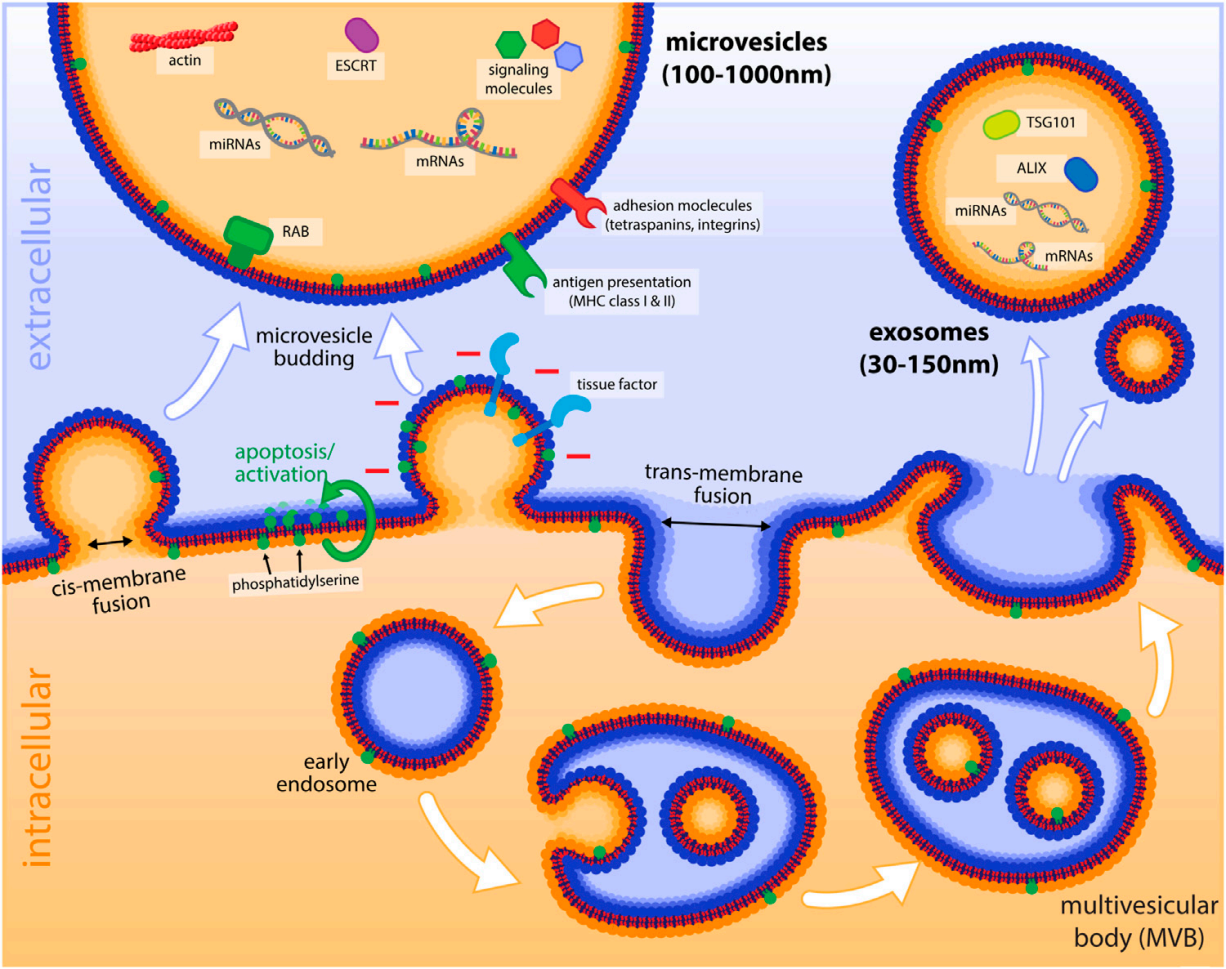
Schematic illustration of microvesicles and endosomes and their formation. Important cargo molecules and markers of microvesicles and exosomes are shown in the lumen of EVs in the extracellular environment. The budding process of microvesicles by cis-membrane fusion is indicated (with the cytoplasmic sides of the membranes getting in first contact) as opposed to the trans-membrane fusion event of endocytosis. The exposure of tissue factor, as well as negatively charged PS (after flipping from the inner leaflet to the outside by cellular activation or apoptosis) is depicted on the budding microvesicle. The lower panel of the figure illustrates the process of endocytosis and the formation of intraluminal vesicles that are found in late endosomes (multivesicular bodies, MVBs) and how these vesicles are released as exosomes, when MVBs fuse with the plasma membrane.

In general, all EVs are released by a membrane fusion event, where the cytosolic sides of two lipid bilayer surfaces get in contact before fusing. This process, which can also be termed as a cis-membrane fusion, differs from fusion events, where the functionally extracellular sides of the bilayer get in contact first (trans-membrane fusion, Figure 1). The latter is realized in endocytosis, when endocytic vesicles are generated by invagination of the plasma membrane, or also during intracellular vesicular transport, when cargo vesicles pinch-off from compartments like the Golgi. Trans-membrane fusion normally requires coat proteins such as clathrin, while cis-membrane fusion events are characterized by the involvement of specific proteins such as SNARE-proteins and small GTPases of the Rab family (Schmid, 2016), which bring the two membrane surfaces in close contact and regulate the specificity of the fusion. This type of fusion occurs, when transport vesicles fuse with their target compartment, or when intracellular compartments with a single lipid bilayer like endosomes fuse with each other. The same sort of cis-membrane fusion is the underlying mechanism, by which patches of endosomal membranes invaginate and bud-off into the lumen of an endosome, thereby forming a multivesicular body (MVB, Figure 1). Furthermore, it is a cis-membrane fusion event when late endosomes or MVBs fuse with the plasma membrane and release their intra-luminal vesicles as exosomes. Similarly, the outward budding of the plasma membrane, followed by contact of the cytoplasmic sides of the membrane and a cis-membrane fusion results in the release of microvesicles, basically representing the opposite of endocytosis.

It is obvious that the membrane topology of EVs is crucial for their biological functions, as the exposure of specific protein domains and lipids, such as the negatively charged phosphatidyl-serine (PS), have a central impact on their interactions with other components of the extracellular environment. Thus, it is extremely important to maintain the correct inside/outside topology and integrity of EVs during their isolation and functional testing. However, many studies, in particular in the early phases of the field, unintentionally overlooked this aspect and applied non-standardized methods of isolation as well as analysis that made it difficult to compare the results of different groups. Consequently, it was a big step forward to initiate guidelines and standards by the International Society for Extracellular Vesicles (Théry et al., 2018). In the following chapter, we discuss some of these aspects in more detail.

### Methods of Preparation of EVs and Their Pitfalls

**FIGURE 2:**
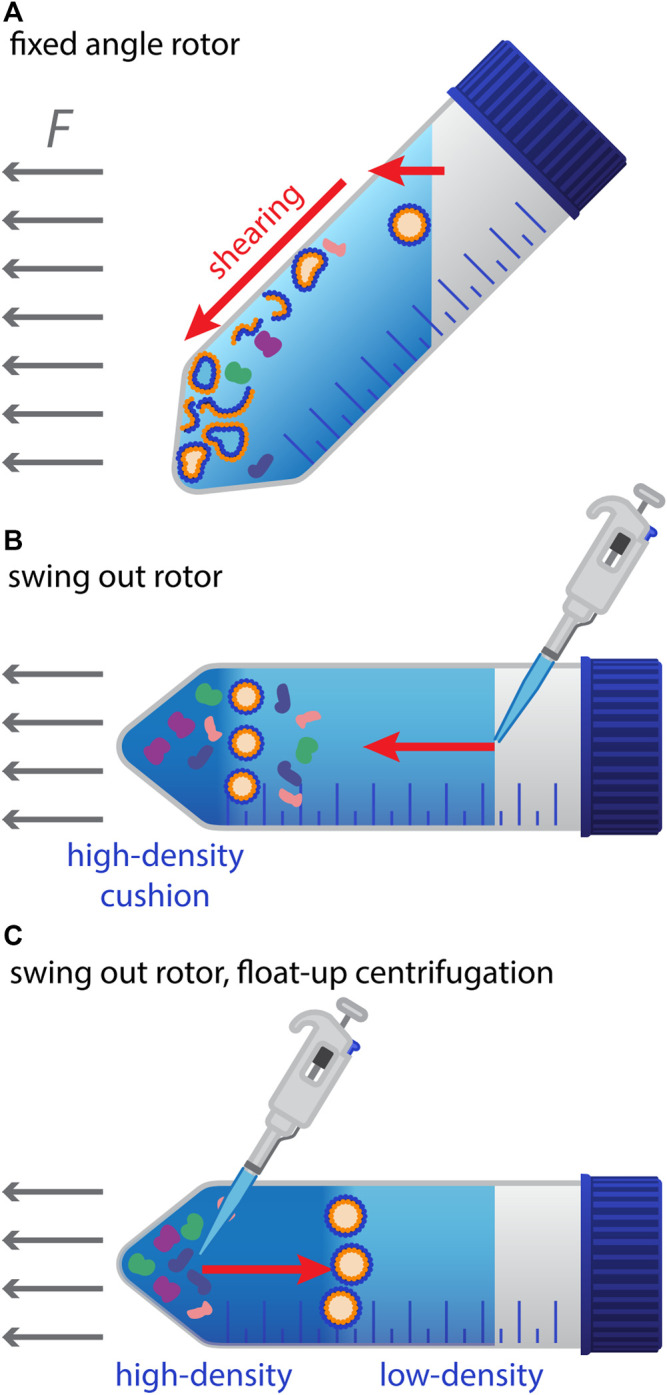
Preparation of EVs by centrifugation and potential pitfalls. **(A)** Illustration of the mechanical stress and shear forces that may damage EVs during high-speed centrifugation with fixed-angle rotors. EVs hitting the tube wall are squeezed and migrate along the wall to the bottom of the tube, where they form a pellet. **(B)** Situation with a swing-out rotor and a high-density cushion on the bottom of the tube to reduce a potential mechanical damage. **(C)** Float-up gradient centrifugation with a swing-out rotor: Suspensions of EVs and potential contaminating particles (e.g., after PEG-mediated precipitation) are applied to the bottom of the tube at a layer of higher density. Upon centrifugation, membranous vesicles such as EVs float up against the centrifugal force vector, until they reach a layer with lower density than their intrinsic one. Protein aggregates and other contaminants are left behind.

The heterogeneity of EVs and their different biological sources (cell cultures, blood, body fluids etc.) resulted in a corresponding variety of methods to isolate, enrich, and analyze them (*see* Table 1). The most frequently used approach for their isolation and enrichment has been and still is sequential centrifugation. Problems with this approach arise from non-standardized rotor types and centrifugation times, resulting in differences in yield and purity of EVs (Cvjetkovic et al., 2014). Ultracentrifugation in itself can lead to aggregation of EVs (Linares et al., 2015), but it is also evident that the type of rotor (fixed-angle or swing-out) can have a significant influence. In most studies, centrifugation is done with conventional fixed-angle rotors. However, this method has some potential drawbacks, as the horizontal forces press the fragile membrane vesicles against the wall of the centrifugation tube, where they are not only squeezed but also forced along the wall down to the site that is most distant from the rotational axis (Figure 2). Obviously, this can lead to significant mechanical stress and shear forces, which can damage the vesicles in several ways. First, they can become leaky (which we often observed in similar-sized endosomes) – and this can occur in a transient manner, as small pores in membranes have the tendency to re-seal. Thereby, they can lose their content fully or partially. Second, various components of the vesicles might be physically damaged. Third, membranous vesicles can break up completely and even re-seal in an inside-out orientation. In line with that, it has been reported for EVs that membrane proteins can occur in a reverse topology with cytosolic domains exposed to the outside (Cvjetkovic et al., 2016). Such an artificial inside-out orientation of EVs caused by their preparation would expose the inner-leaflet PS to the outside and would lead to a false-positive binding of Annexin V or lactadherin to the vesicles. The problem of the shear forces can be reduced by using swing-out rotors. Here the particles move along the axis until they reach the bottom of the tube. Yet, this approach does not avoid mechanical stress completely as the vesicles are still pressed against the bottom. The latter might be reduced by using a liquid high-density cushion, such as a sucrose-solution at the bottom of the tube. Finally, the principle of float-up gradient centrifugation (Burdon et al., 1988) can be applied, where membranous vesicles are applied to a centrifugation tube at a layer of higher density than their inherent buoyant density, which is overlaid with layers of lower density. During centrifugation in a swing-out rotor, the vesicles (but not free proteins or protein aggregates) will float up against the direction of the centrifugation force through the layers of higher density until they reach a layer, which has a lower density than their intrinsic one. There they will accumulate at the interface of the two layers, which can even have an enrichment effect (Figure 2). Such float-up gradient approaches have also been applied for EVs (Iwai et al., 2016; Li et al., 2018). An advantage of these flotation gradient techniques is that they have a significant purification effect and separate EVs from contaminating protein aggregates and lipoproteins. The pitfalls and issues of classical differential (ultra-) centrifugation using fixed-angle rotors have been described in several reports (Cvjetkovic et al., 2014; Linares et al., 2015; Livshits et al., 2015; Söderström et al., 2016). However, it still seems to be the most widely used method to prepare EV samples. Nevertheless, additional, alternative methods have been established to purify these vesicles with the aim to maintain their integrity. These include ultrafiltration and size-exclusion chromatography (Nordin et al., 2015) and are sometimes combined with polymeric precipitation using polyethylene glycol, PEG (Szatanek et al., 2015). More recently, a method has been reported, which combines PEG precipitation with Nycodenz (iohexol) gradient centrifugation followed by size-exclusion chromatography (Zhang et al., 2020), which seems to solve some of the problems that are associated with the traditional ultracentrifugation technique. Another word of caution has to be expressed with respect to potential influences of buffers, in which EVs are prepared and resuspended for further analysis. It has been demonstrated, for instance, that the number of EVs can increase with time, after the initial sampling, dependent on the buffer in which the samples are stored, with a significant increase of EV numbers in heparin- and citrate-containing buffers as compared to EDTA-buffers (Fendl et al., 2016). Moreover, it could be shown that storage of samples in PBS (in contrast to saline) can result in artefacts by creating EV-like particles, which are also positive for EV-markers in subsequent flow cytometry analyses (Xin et al., 2017).

**TABLE 1:** Methods of EV isolation, preparation and purification and their pitfalls.

Method	Strengths	Pitfalls	References
Sequential centrifugation with fixed angle rotors	• easy to perform with standard equipment	• shear forces can damage EVs, lead to leakiness and loss of cargo, and generation of inside-out vesicles (thereby false-positive exposure of phosphatidylserine)	(Cvjetkovic et al., 2014; Linares et al., 2015; Livshits et al., 2015; Szatanek et al., 2015; Théry et al., 2018; Lee et al., 2019)

	• fairly fast	• ultracentrifugation as final enrichment can lead to aggregation of EVs	
	• enrichment can be achieved by final ultracentrifugation		
Sequential centrifugation with swing-out rotors	• easy to perform with standard equipment	• centrifugation without a high-density bottom layer can still lead to EV damage	(Szatanek et al., 2015; Li et al., 2018; Théry et al., 2018; Lee et al., 2019)
	• fairly fast	• A sucrose solution as bottom cushion has strong osmotic strength, which might affect EVs	
	• enrichment can be achieved by final ultracentrifugation		
Float-up gradient centrifugation	• still easy to perform with standard equipment	• The gradient material might have unknown influences on EV functions, thus inert gradient substances should be used	(Iwai et al., 2016; Li et al., 2018; Théry et al., 2018; Lee et al., 2019; Zhang et al., 2020)
	• enrichment at the interface of two layers with different densities is possible		
	• Purification effect (from associated proteins or aggregates) by the float-up process		
Precipitation with polymers such as PEG	• Fast method	• Precipitation alone results in low purity and contamination with lipoprotein particles and should therefore be combined with other methods	(Szatanek et al., 2015; Théry et al., 2018; Zhang et al., 2020)
	• Kits are available		
Size-exclusion chromatography	• mild purification with hardly any damage of EVs	• Rather time-consuming and equipment not lab standard	(Nordin et al., 2015; Szatanek et al., 2015; Théry et al., 2018; Lee et al., 2019; Zhang et al., 2020)

### Methods to Analyze EVs and Their Limitations

For reasons of practicability and availability, microvesicles and other larger EVs are often investigated and analyzed by flow cytometry. However, standard cytometry equipment only allows analysis down to about 300 nm diameter particles, which excludes a high number of EVs. Specific instruments can get down to about 100 nm, which would comprise the entire size range that has classically been defined for microvesicles but would still omit most exosomes. For that reason, complementary and alternative methods are in use, such as pelleting EVs with ultracentrifugation at >100,000 g for 1 h or longer (keeping in mind the resulting limitations discussed in section 1.1), followed by biochemical analysis, such as SDS-PAGE and Western Blot testing of known markers of EVs as well as frequent contaminants (like apolipoproteins). Biochemical analysis of EVs can include degradation of presumable intraluminal cargo (like miRNAs) to check for contamination of EV preparations with inside-out vesicles. Similarly, binding of antibodies to extravesicular domains of proteins followed by cytometry or microscopy can be applied to test for correct orientation of EVs. Many groups also use electron microscopy (EM) to check for integrity and purity of EVs, as contaminating lipoprotein particles appear differently in EM. In this case, cryo-EM was reported to outperform transmission-EM, as the latter can often lead to artefacts caused by fixation and contrast agents (Cizmar and Yuana, 2017).

A technique, which found increasing application in the field, is nanoparticle tracking analysis (NTA), which basically measures the Brownian motion of particles detected by scattered light or fluorescence (Saveyn et al., 2010). This technique can determine much smaller vesicles than flow cytometry and has the advantage to measure the concentration of particles, as well as their size distribution. However, it does not differentiate between EVs and lipoprotein particles and therefore has to be combined with additional methods to prove the purity of an EV preparation. Furthermore, results can vary significantly between different machines and labs and optimized instruments settings have to be applied to obtain consistent and robust data (Vestad et al., 2017; Bachurski et al., 2019). Direct comparisons between flow cytometry and NTA for platelet-derived EVs demonstrated that only a smaller fraction of particles detected by NTA in light scatter mode are really membranous vesicles, and that only about 10% of the particles detected in fluorescence mode-NTA were PS-positive, as compared to about 60% for flow cytometry (George et al., 2021). This supports the notion that flow cytometry detects only larger EVs, which have a higher fraction of PS-positive vesicles. An overview of analysis techniques is shown in Table 2.

**TABLE 2:** Methods of EV analysis and their limitations.

Method	Strengths	Pitfalls/Limitations	References
Flow cytometry	• Fast method with single particle resolution and excellent statistics	• smaller EVs are not detectable (lower limit: about 100–300 nm)	(Ayers et al., 2011; van der Pol et al., 2014; Fendl et al., 2016; Théry et al., 2018; George et al., 2021)

	• Fluorescent antibodies or lipophilic dyes can be applied for specific detection	• Equipment not available in all labs	

	• Combination with degradation approaches or antibodies binding to extravesicular domains allows detection of inside-out EVs	• Results may vary between labs (dependent on instrument settings)	

	• Combined with flow sorting it can be used to purify subpopulations for further differential analysis	• Proper gating strategy is important to analyze the correct set of particles (raw data and gating are often not shown in publications for all results)	
Nanoparticle tracking analysis (NTA)	• Precise determination of size range and concentration is possible	• Light-scattering mode does not discriminate between membranous vesicles and protein aggregates or lipoproteins	(Saveyn et al., 2010; van der Pol et al., 2014; Holnthoner et al., 2017; Vestad et al., 2017; Théry et al., 2018; Bachurski et al., 2019; George et al., 2021)
	• Fluorescence mode particle tracking allows more specific detection of EVs		
Biochemical analyses (SDS-PAGE, Western Blots, etc.)	• Allow more specific analysis of EV components	• Require a correct purification of EVs	(Thomas et al., 2009; Iwai et al., 2016; Fendl et al., 2018; Théry et al., 2018; Lee et al., 2019; Tripisciano et al., 2020; Zhang et al., 2020)
	• Can be used to determine contaminants and the purity of EV preparation		
Next Generation sequencing approaches or proteomics	• Unbiased and integrative analysis of EV constituents and cargo	• Expensive and requires access to core facilities of sequencing or proteomics	(Laroche et al., 2017; Théry et al., 2018; Veziroglu and Mias, 2020; Xu et al., 2020; Hildebrandt et al., 2021)
	• Can be used for differential analysis (e.g., disease versus control)	• bulk sequencing or proteomics does not allow to determine subpopulations of EVs (has no single particle resolution)	
Electron microscopy	• Provides high resolution images of EVs and size information	• low-throughput and tedious method	(Arraud et al., 2014; van der Pol et al., 2014; Cizmar and Yuana, 2017; Théry et al., 2018)
	• can differentiate between EVs and lipoprotein particles or protein aggregates	• equipment often not available	
		• mostly unspecific as immuno-EM is difficult and sometimes impossible	

### Functional Assays to Characterize EVs

EVs are commonly characterized by measuring their composition. Dependent on the cellular origin and (patho-)physiological state, EVs carry a multitude of molecules inside or on their surface, including RNA, DNA, proteins, lipids and metabolites. Thorough analysis of their constituents, e.g., by next generation sequencing, Western Blot, ELISA or different omics approaches, can give insight into their pleiotropic responses. In addition, functional assays are applied to characterize EVs, which have been recently reviewed in (Coumans et al., 2017; Théry et al., 2018). A short summary of these assays and references for them are included in Table 3. Their validity is dependent on the use of specific antibodies and the development and use of reference materials and controls as well as standardized collection, handling, and isolation of EVs for data comparison.

**TABLE 3:** Functional assays for EVs.

General aspects			Reviewed in (Coumans et al., 2017; Théry et al., 2018)
**Biological function**	**Methods**	**Pitfalls/Limitations**	**References**
**Coagulatory functions**			
Clotting time	• Measuring clotting time of plasma upon FX activation to determine EV-PS activity	• Restricted to plasma samples	(Exner et al., 2003; Luddington and Baglin, 2004)
Thrombin generation	• Measuring thrombin generation after capture of EV-PS on annexin-V coated ELISA plates upon addition of TF and phospholipids	• Requires an inhibitor of contact activation, e.g., CTI	(Hemker et al., 2002; Jy et al., 2004)
	• Measuring coagulant EV-TF in terms of thrombin, fibrin of FXa generation	• Use of specific antibodies to block TF coagulant activity	(Hellum et al., 2012; Thaler et al., 2012; Thaler et al., 2013; Tatsumi et al., 2014)
	• TF-dependent FXa generation upon addition of FVII and phospholipids	• PS-quantification only when PS source is restricted to EVs	Summarized in (Agouti et al., 2015; Hisada et al., 2016; Hisada and Mackman, 2019)
	• Fibrin generation in plasma determines EV’s PS and TF activity	• Requires concentration of plasma EVs by centrifugation, but concentration and isolation of EVs contributes to poor reproducibility of the respective functional test	Berckmans et al. (2011)
**Fibrinolysis**			
Plasmin generation	• Measurement of plasmin generation using plasmin-selective chromogenic or fluorogenic substrates	• No standards available	(Lacroix et al., 2007; Miszta et al., 2020)
		• Needs controls specific for plasmin generation, such as α2-antiplasmin or an inhibitory antibody against urokinase	
**Cellular functions (including EC-functions)**			
Migration	• Effects of EVs on trans-well migration and wound healing models (e.g., scratch assays)	• Cell culture or organoid models might not reflect the *in vivo* situation sufficiently	(Silva et al., 2017; Kriebel et al., 2018)
Proliferation	• Effects of EVs on cell numbers		Zhong et al. (2020)
	• DNA-synthesis (BrdU incorporation etc.)		
Formation of spheroids and sprouts	• Sprouting of ECs from beads		Hood et al. (2009)
	• Formation of 3D-organoids (e.g., of cancers cells)		
TEER (Transendothelial Electrical Resistance)	• TEER measurement using HUVECs or isolated primary endothelial cells		Dean et al. (2009)
Tube formation			Skog et al. (2008)

### Cellular Sources of EVs in Blood

Extracellular vesicles play crucial roles in intercellular communication and are released under physiological and pathological conditions from various cell types, including platelets, red blood cells, lymphocytes, dendritic cells, hematopoietic cells, endothelial cells or cancer cells (van Niel et al., 2018).

Cellular sources of EVs in the blood are primarily distinguished based on their surface antigens. CD41 is a common marker for platelet- or megakaryocyte-derived EVs (Flaumenhaft et al., 2009; French et al., 2020). **Megakaryocytes** in the bone marrow constantly release EVs into the blood stream. Therefore, these EVs dominate in healthy individuals, which is also reflected by the fact that they were initially described as “platelet-dust” (Wolf, 1967). **Platelet-derived EVs (PEVs)** are only released upon stimulation. They expose the platelet activation markers P-selectin and PS, which are both absent on megakaryocytic EVs. The exact concentration of PEVs in healthy plasma is still controversially discussed with concentrations specified as low as 200 up to 10^9^ EVs/µl (Arraud et al., 2014). This is most likely attributed to low sensitivity of the detection method or false co-detection of contaminants (Arraud et al., 2014; Johnsen et al., 2019). Despite this controversy, it is clear that EVs derived from platelets and megakaryocytes represent the majority in the circulation (Flaumenhaft et al., 2009; Aatonen et al., 2014; Arraud et al., 2014).

**Red blood cells (RBCs)** are the most abundant cell type in the blood. EVs derived from RBCs participate in physiological blood clotting by activating the intrinsic coagulation pathway *via* FXIIa and FXIa leading to thrombin generation (Van Der Meijden et al., 2012; Rubin et al., 2013). Conversely, RBC EVs can also interact with protein S and thereby support the anticoagulant reaction mediated by aPC (activated protein C) (Livaja Koshiar et al., 2014). Further, RBC EVs can contribute to dysregulation of hemostasis and demonstrate pro-coagulant effects in several disease states and can interact with the endothelium. EVs released from fresh or stored RBCs have pro-inflammatory properties and are closely related to the immune and inflammatory responses of blood transfusions, where they might contribute to adverse outcomes (Straat et al., 2016; Crawford et al., 2019).

Besides RBCs and platelets, also **leukocytes** contribute to EVs in the circulation. The main function of leukocyte derived EVs is to induce immune responses, to recognize and eliminate pathogenic or harmful substances, and to participate in inflammatory processes. Neutrophil-derived EVs can exert both pro- and anti-inflammatory effects on neighboring cells (Kolonics et al., 2020; Kolonics et al., 2021), depending on the environmental conditions during EV generation. Cell surface proteins of neutrophil-derived EVs mediate their adherence to monocytes and endothelial cells inducing downstream signaling pathways (Danesh et al., 2018). An example of an anti-inflammatory role of neutrophil-derived EVs is the potential release of TGF-β1, which inhibits the inflammatory response of macrophages to zymosan and LPS (Gasser and Schifferli, 2004). Contrary, EVs released from neutrophils upon bacterial infections in mice promote platelet activation (Letsiou et al., 2021) and enable platelet-mediated innate immune responses by inducing robust inflammation and pathogen clearance (Rossaint et al., 2016).

**Endothelial cell-derived EVs** (EC-EVs, markers: CD31^+^, CD144^+^) play an important role in intercellular communication under homeostatic and pathological conditions, e.g., under inflammation (Yamamoto et al., 2015), and correlate with disease severity. EC-EVs expressing tissue factor (TF) (Zwicker et al., 2011) exhibit procoagulant activity as shown *in vitro* and in animal studies (Holnthoner et al., 2017). EC-EVs have recently attracted attention as mediators and potential therapeutic targets especially in respiratory diseases, such as asthma or COPD. They are reviewed in more detail elsewhere (Fujimoto et al., 2020; Desideri et al., 2021).

### More Details on Platelet-Derived Extracellular Vesicles (PEV)

Platelets are small anucleate cells derived from their precursors, megakaryocytes, in the bone marrow. They are primarily known for their role in hemostasis and vascular integrity. Platelets generate and release EVs upon activation by a variety of agonists. Previously, it was hypothesized that PEVs released from activated platelets or platelet-rich thrombi may reflect developing arterial thrombosis and that therefore, the role of platelets and PEVs in hemostasis may be redundant (Puhm et al., 2021). However, due to their complex set of cargo, PEVs participate in diverse biological processes such as angiogenesis, cancer, cardiovascular diseases, or inflammation. Historically, two different types of platelet-EVs were discriminated according to their size. Platelet-MVs (100–1000 nm in diameter) usually expose PS on their surface (when generated from activated platelets) and express GPIbα, αIIbβ3 and P-selectin (Heijnen et al., 1999). Smaller platelet-exosomes (40–100 nm) expose CD63, do not bind to FX and prothrombin, and are difficult to detect by flow cytometry due to their size (Heijnen et al., 1999). Accordingly, the procoagulant activity of PEVs was mainly ascribed to microvesicles. Mallat and others associated procoagulant activity of PEVs predominantly with TF (Mallat et al., 1999). Although the occurrence of this coagulation factor on platelets is still questioned (Østerud and Bouchard, 2019), it has been proposed that platelets can acquire TF from TF-bearing EVs originating from other cell types through fusion in a PSGL-1-dependent way (Del Conde et al., 2005).

Besides TF, a major procoagulant effect of PEVs is apparently the exposure of negatively charged phospholipids (e.g., PS), which create complex bonds with Ca^2+^ ions, that are further binding the (Vitamin K-dependent) γ-carboxy groups of coagulation factors. In this way, components of the coagulation cascade are concentrated on the surface of negatively charged membranes, which results in a drastic increase in the rate of proteolytic conversion and activation of coagulation factors. Thus, PS-exposing PEVs provide a surface for assembly of tenase and prothrombinase complexes, thereby being directly involved in coagulation (Owens and Mackman, 2011), which implies a causal relationship between increased PEV concentrations and thrombosis. In general, it can be stated that the release of PEVs contributes to the increase in membrane surface area together with cytoskeletal rearrangement and the formation of filopodia upon platelet activation.

The procoagulant activity of PEVs depends on the underlying trigger leading to their release from platelets (Sims et al., 1989). Highly procoagulant PEVs are formed after platelet activation with a combined stimulus of collagen and thrombin, complement C5b-9 or a calcium ionophore, whereas the procoagulant activity of PEVs is lower upon activation with ADP, epinephrin or thrombin alone (Sims et al., 1989; Zwaal et al., 1992). In line with that, PEV populations are highly heterogeneous (French et al., 2020). Further diversity arises from differences in PEV content as well as PEV quantity, which is increased in a variety of diseases associated with chronic inflammation or ongoing platelet activation, such as cardiovascular diseases, stroke, sepsis, multiple types of cancers or autoimmune diseases (such as multiple sclerosis, or rheumatic arthritis) (Abrams et al., 1990; Varon and Shai, 2009; Chiva-Blanch et al., 2017; Gasecka et al., 2017). Disease-dependent changes in the biochemical composition of PEV may be useful for differential diagnosis given the ease of sampling. While PEVs had primarily been associated with procoagulant activity, another study gave first hints that PEVs may exert both pro- and anti-coagulatory effects, as some PEVs preparations were shown to inactivate Factor Va (Tans et al., 1991) through binding of Protein S to the coagulation inhibitor protein C (Dahlbaeck et al., 1992). Besides their effects on hemostasis, PEVs can exert immune-modulatory roles like platelets themselves. It has been shown for example, that they can inhibit IL-17 and IFN-γ production by Tregs in a P-selectin dependent manner (Chimen et al., 2020).

From the observations so far, it seems likely that half-life and clearance mechanisms of (P)EVs are dependent on pathophysiological conditions, such as the presence of inflammation. It remains to be established whether increased concentrations of PEVs in thrombosis are due to increased formation, less efficient clearance, or a combination of both. An important aspect of PEVs and other EVs in contrast to platelets is that their small size allows them to cross tissue barriers, for instance through fenestrated endothelium, whose pores are too small for platelets but large enough for EVs. These pores can have diameters of about 100–200 nm and sometimes reach even 1 µm. They occur in sinusoidal vascular beds of organs with prominent filtration or transendothelial transport processes, such as kidney, spleen and liver, with the latter exhibiting the space of Disse between endothelium and hepatocytes (Krams and Bäck, 2017). It is important to note that EVs not only enter these tissues from the blood through those pores, but also can be released by the underlying tissue and find their way into the circulation. This may also be used for diagnostic purposes, for example to monitor pathological liver states (Thietart and Rautou, 2020; Marcoux et al., 2021). Furthermore, EVs can also enter other anatomical sites such as lymph (Milasan et al., 2016; Tessandier et al., 2020), bone marrow (French et al., 2020) or even synovial fluid (Boilard et al., 2010; Puhm et al., 2021). By that, they can affect many different cell types and contribute to distant cell-cell communication beyond the circulation (Puhm et al., 2021). Recently, Tessandier et al. demonstrated that platelet-derived serotonin stimulated the transit of PEVs to lymph nodes, thereby promoting rheumatoid arthritis (Tessandier et al., 2020). Besides delivering intravesicular cargo to target cells, (P)EVs sometimes contribute to intercellular communication, by transferring surface proteins (such as C3, TF, or fibrinogen) in a piggyback manner to other cells, thereby creating interactive membranes that contribute to inflammation, thrombosis and cancer (Palviainen et al., 2020).

In conclusion, it can be stated that PEVs share many of the features and functions of platelets, which explains why they had originally been designated as “platelet dust” (Wolf, 1967). Upon platelet activation, newly released PEVs contribute to the expansion of “reaction surface” in parallel to the membrane increase caused by degranulation and by cellular protrusions formed *via* cytoskeletal remodeling. Beyond that they contribute to the diversity of platelet function by adding another layer of complexity through their roles as intercellular communicators transferring a great variety of cargo molecules to different, even distant target cells.

## Roles of EVs in Inflammation, Cancer and Thrombosis

### Blood-Derived EVs in Inflammation

EVs play a role in diseases with acute inflammation (e.g., sepsis, *acute respiratory distress syndrome*, ARDS) (Sanwlani and Gangoda, 2021) as well as in chronic inflammatory conditions (e.g. asthma, chronic obstructive pulmonary disease, COPD, obesity) (Campello et al., 2016a; Alashkar Alhamwe et al., 2021; Purghè et al., 2021; Sanwlani and Gangoda, 2021). An example of a crucial chronic inflammatory process is atherosclerosis, the chronic inflammation of arteries, which is driven by LDL-cholesterol and immune cell infiltration. EVs derived from blood and vascular cells participate in the progression of atherosclerosis in several ways. Together with LDL-cholesterol, they promote inflammation, vascular dysfunction, leukocyte adhesion and tissue remodeling, leading to increased thrombotic risks (Owens and Mackman, 2011). Neutrophil-derived EVs, for example, contribute to atherogenesis through delivery of miR-155 to disease-prone regions enhancing NF-kB signaling and macrophage content (Gomez et al., 2020). EVs exposing TF and PS, which are retained in the plaque, account for the procoagulant activity of the lipid core, promote coagulation, and thereby increase plaque vulnerability (Chiva-Blanch and Badimon, 2019). Furthermore, PS-positive EVs can induce apoptosis and modulate gene expression (Del Conde et al., 2005). The function of different cellular EVs in atherosclerosis was recently reviewed in greater detail (Chiva-Blanch and Badimon, 2019).

EVs of platelets, RBCs and endothelial cells mediate inflammatory responses by interaction with PBMCs (peripheral blood mononuclear cells), mostly monocytes, causing the secretion of proinflammatory chemokines and cytokines, and leading to an increased survival of PBMCs (Danesh et al., 2014). Endothelial EVs, for example, were shown to correlate directly with IL-6 *in vitro* and *in vivo* (Curtis et al., 2009). RBC-derived EVs exhibit immune-inflammatory functions by modulating the activity of T and B cells. They can inhibit for example B cell activity after LPS stimulation by inhibition of the NF-kB pathway (Gao et al., 2020). On the other hand, RBC-EVs are able to bind to monocytes, thereby inducing the release of proinflammatory cytokines (IL-1, IL-6, TNF-α) and chemokines (*macrophage-derived chemokine,* MDC, and *macrophage inflammatory protein 1a,* MIP-1a), which induce T cell proliferation (Danesh et al., 2014). Besides inducing the release of pro-inflammatory cytokines by monocytes, EVs can also contain these mediators as cargo (including IL-12, IL-15, IL-17, and IFN-γ) and transfer them to other cells at early stages of inflammation (Sanwlani and Gangoda, 2021). Similarly, PEVs carry IL-1β, lipid mediators and DAMPs (e.g. HMGB1) (Puhm et al., 2021) and can modify CRP (*C-reactive protein*) into its pro-inflammatory form (Braig et al., 2017). An inflammatory role has also been shown for EVs transferring CD40L, which binds to monocytic CD40 and triggers the activation of NF-kB *via* the alternative pathway, causing the release of inflammatory mediators from monocytes (Bei et al., 2016). On the other hand, miR-125b-5p in RBC-EVs negatively regulates IL-1β induced inflammatory gene expression by targeting the TRAF6/MAPKs/NF-kB pathway (Rasheed et al., 2019).

Beside their role in the acute or early phase of inflammation, EVs also contribute to its resolution by carrying anti-inflammatory cytokines, such IL-4 or IL-10 (Gao et al., 2019) or by providing 12-lipoxygenase to mast cells which enhances the production of lipoxin A4 (Tang et al., 2010). PEVs can further polarize macrophages to an anti-inflammatory state or regulate adaptive immunity by inducing anti-inflammatory signaling in plasmacytoid dendritic cells (Sadallah et al., 2011).

### EVs in Cancer

The number of EVs is increased about twofold in the blood circulation of cancer patients reaching a sum of about 4 × 10^15^ (Kalluri, 2016). The reason for this increased occurrence is still not clarified in detail, but it is known that ESCRT genes, as well as other proteins and lipids that play a role in EV formation, are elevated in various tumors (Kilinc et al., 2021). While the biological roles of EVs are in general very diverse and complex, some of their effects on cancer development and progression have already been elucidated. Importantly, EVs released by cancer cells can fuse with other cells in the vicinity and thereby transfer their cargo, which can then exert a variety of effects on the recipient cells. Material that can be passed on includes proteins with pro-tumorigenic roles such as growth factors or expression products of oncogenes (e.g. KRAS, or EGFR variants), but also mRNAs, as well as miRNAs, which influence the cellular program of target cells on the transcriptional and post-transcriptional level (Bebelman et al., 2018). Furthermore, EVs can influence the tumor microenvironment in a way, which fosters further tumor growth. They can for example influence the fate of neighboring cells towards cancer-associated fibroblasts, which create a favorable niche for the tumor, or they can contribute to epithelial-mesenchymal transition (EMT). EVs can also promote angiogenesis and contribute to remodeling of the extracellular matrix (ECM) in a way, which supports metastasis from the primary tumor site, as well as the generation of pre-metastatic niches at distant sites, where cancer cells can settle. Besides, EVs can have multiple effects on immune cells, with both pro- and anti-tumorigenic consequences. On one hand, tumor-derived EVs carrying peptide-loaded MHC-I molecules can activate T-cell responses either directly or by cross-presentation *via* dendritic cells (DCs) and they can also activate macrophages or NK cells. On the other hand, exosomes are able to suppress anti-tumor immunity and immune surveillance by various means. TGFβ-containing EVs can downregulate cytotoxic T-cell function and increase immune-response dampening regulatory T-cells. Exosomes carrying FasL or TRAIL can induce apoptosis of activated T-cells and furthermore EVs can expose PD-L1 and thereby inhibit T-cell mediated immunity against cancers. EVs have the potential to affect myeloid cells by suppressing DC differentiation and can enhance immune-suppressive functions of myeloid-derived suppressor cells (MDSCs). Moreover, it was reported that EVs released from tumor cells promoted macrophage polarization towards the M2 phenotype, which exerts rather wound healing than defense functions and is considered tumor-promoting (Kalluri, 2016; Bebelman et al., 2018; Mashouri et al., 2019; Zhang and Yu, 2019).

Apart from these direct effects of EVs on cancer development and progression, they can have important diagnostic roles for the non-invasive detection of cancer *via* liquid biopsies using blood, urine or other body fluids, in which for example cancer-EV specific miRNAs or mRNAs are quantified. In combination with next-generation sequencing approaches, these samples can also give information on mutational signatures of cancers with the advantage that heterogenous or multi-focal tumor tissue, which is difficult to sample by standard biopsies can be analyzed and stratified altogether in one sample. While this field seems to have a great potential, it still needs better standardization and proof *via* larger patient cohorts (Kalluri, 2016; Möller and Lobb, 2020).

Further potential applications of EVs lie in cancer therapy. The ability of EVs to fuse with target cells in an efficient and sometimes specific manner renders them ideal candidates for the delivery of therapeutics, where they are often more effective than liposomes or lipid-based nanoparticles. Besides chemical drugs, they are also capable of delivering siRNAs or shRNAs that target oncogenes or mutations. In that context, CD47-positive exosomes are particularly interesting, as they are less taken up by macrophages due to their “don’t eat me signal” and therefore seem superior to normal lipid particles. Interestingly, EVs can be even prepared from plant cells, which would allow an easier scale-up to larger quantities (Kalluri, 2016; Zhang and Yu, 2019; Möller and Lobb, 2020). Apart from the use as delivery vehicles, EVs are also increasingly recognized as candidates for anti-cancer vaccines, based on their ability to modulate the immune response. For that purpose, exosomes can be used to present tumor-antigens so that cytotoxic T-cells are activated, or DCs may be used to generate exosomes that express MHC I and II and thereby induce anti-tumor immunity (Kalluri, 2016; Zhang and Yu, 2019; Möller and Lobb, 2020).

### EVs in Arterial Thrombosis

Although EVs are elevated in variety of thrombotic diseases, it remains difficult to pinpoint the role of EVs in thrombosis in detail.

To analyze the role of EVs in arterial thrombosis, previous studies used a FeCl_3_-induced or photochemical injury of the mouse carotid artery (Wang et al., 2009; Kretz et al., 2010). Here, thrombosis is mainly driven by TF derived from the vessel wall (Day et al., 2005), since this injury type causes disruption of the endothelium. Mice injected with TF-positive EVs derived from monocytes showed increased fibrin accumulation implying that they contribute to thrombosis. Of note, these experiments were conducted in healthy mice with low levels of circulating TF+ EVs and the amount of TF+ EVs injected in the study may exceed those found in disease states. A second, frequently used model to study arterial thrombus formation is the laser injury model, where a small arteriole, usually of the cremaster muscle, is injured with a focused laser beam. This model may be better suitable to study the role of EVs in thrombosis since the injury per se causes less injury to the vessel wall (Rosen et al., 2001). Here, a rapid accumulation of TF staining upstream of the thrombus was observed (Gross et al., 2005), indicative of TF+ EV recruitment to the thrombus (Falati et al., 2002), as well as an incorporation of exogenously added TF+ EVs into the developing thrombus. This incorporation is dependent on PSGL-1 on EVs and platelet P-selectin (Falati et al., 2003). Using low TF mice, the cellular source of EV contributing to arterial thrombosis was pinpointed to neutrophil-derived TF+ EVs binding to endothelial cells *via* LFA-1 ICAM-1 interactions (Darbousset et al., 2012).

### EVs in Venous Thrombosis

While arterial thrombosis occurs mainly after rupture or erosion of atherosclerotic lesions and is mainly characterized by a platelet-rich thrombus, venous thrombosis happens predominantly in regions of blood stasis and is characterized by a fibrin-, neutrophil- and red blood cell-rich thrombus.

Ligation of the interior vena cava (IVC) is the standard model to study venous thrombosis. Similar to arterial thrombosis in the cremaster, a role for P-selectin and leukocyte-derived EVs was shown in venous thrombosis using the IVC ligation model (Myers et al., 2003) with EVs being recruited to the thrombus. While thrombus weight correlated positively with PEVs, a negative correlation was shown for leukocyte-derived EVs (Ramacciotti et al., 2009). TF-positive EVs in venous thrombosis were studied in several models (Himber et al., 2003; Myers et al., 2003; Szema et al., 2005; Hawley et al., 2010) with an overall correlation of TF with increased EV concentration and increased thrombus mass. In line with that, mice deficient for the NF-kB transcription factor p50, which have a blunted TF expression, showed reduced venous thrombosis (Li et al., 2009). In carriers of factor V Leiden mutation, circulating EVs, especially PS-positive EVs, contribute to the development of venous thrombosis (Campello et al., 2012).

### EVs and Coagulation

Generally, the plasmatic coagulation cascade can be triggered by a so-called extrinsic and an intrinsic pathway. The first is physiologically triggered by injury of the endothelium and exposure of subendothelial TF. This initiates a complex cascade of enzyme activations, involving factors VII, VIII, IX and X that generates a significant amount of active thrombin *via* several amplification loops. Thrombin then converts fibrinogen to fibrin, which polymerizes and is finally cross-linked to form a stable fibrin network (Furie and Furie, 1988). The intrinsic activation pathway (or contact pathway) can be triggered by negative charged surfaces, like those exposed by neutrophil-extracellular traps (NETs) and involves factors XII and XI, and merges with the other pathway at the level of factor IX. In parallel to the plasmatic coagulation cascade, platelets adhere to the injured site, are activated, and aggregate through interactions of platelet receptors with extracellular ligands. Both the plasmatic cascade and platelet activation stimulate each other as thrombin activates platelets *via* PAR receptors (protease-activated receptors) and activated platelets release fibrinogen, as well as factors V and VIII *via* their α-granules. Finally, platelets contract to form a densely packed thrombus (Krishnaswamy et al., 1993; Swieringa et al., 2018).

Platelets interact with the coagulation system in a multitude of ways, not only during thrombus formation, but also in specific areas within a formed thrombus (Swieringa et al., 2018). In the thrombus core, procoagulant, PS-exposing platelets increase the formation of tenase and prothrombinase complexes *via* the negatively charged phospholipid surface. This amplifies thrombin generation and links PS-exposure to platelet activation and coagulation (Zwaal and Schroit, 1997). Multiple enforcement loops exist both in the coagulation cascade and in platelet activation leading to a fast and efficient formation of a stable fibrin- and platelet-rich thrombus (Macfarlane, 1964; Versteeg et al., 2013).

While platelets and PEVs provide the majority of negatively charged surfaces under physiological conditions, and perivascular TF is required to initiate coagulation, the situation is different under pathological conditions. In disease states such as sepsis or cancer, a significant amount of TF can be found in circulation, mostly on the surface of microvesicles, which primes the blood for coagulation (Wolf, 1967). It has been proposed that the TF on MVs is predominantly inactive, residing on the surface in a so-called encrypted conformation, but that it can be activated by a conversion of disulfide bonds, which can be catalyzed by protein disulfide isomerase (PDI), explaining the procoagulant role of this enzyme in the circulation (Versteeg and Ruf, 2011; Stopa and Zwicker, 2018).

### Combinatorial Roles of EVs in Inflammation, Cancer and Thrombosis

#### EVs in Cancer-Associated Thrombosis

The association between cancer and thrombosis was first described by Bouillard (Bouillard et al., 1823) and Trousseau (Trousseau, 1865). Thrombotic complications in cancer vary from arterial or venous thromboembolism (VTE) to disseminated intravascular coagulation (Eichinger, 2016). Since the first description, multiple clinical studies provided evidence that patients suffering from VTE (comprising deep vein thrombosis and pulmonary embolism) have a higher risk for cancer (Murchison et al., 2004) and conversely that cancer patients also have an elevated risk for VTE (Blom et al., 2005; Campello et al., 2016b). Despite novel cancer therapies and improved survival, the incidence of VTE in cancer patients is still increasing steadily (Mulder et al., 2021), and epidemiology, pathophysiology, as well as risk factors of cancer-associated thrombosis have been described in several reviews (Blom et al., 2005; Noble and Pasi, 2010; Eichinger, 2016; Abdol Razak et al., 2018).

The roles of EVs in cancer-associated thrombosis were studied in both mice and men (Dvorak et al., 1981; Wang et al., 2012; Rautou and Mackman, 2013; Geddings et al., 2016). Earlier animal studies demonstrated the release of tumor-derived EVs from a variety of tumors *in vivo* and confirmed the exposure of procoagulant TF on EVs in various *in vitro* assays (Davila et al., 2008; Wang et al., 2012). Tumor-derived TF+ EVs were shown to activate coagulation and enhance thrombosis linking cancer and thrombotic risk (Campello et al., 2016b). The effect of these EVs on thrombosis was further underscored by a study from Thomas *et al.* (Thomas et al., 2009) demonstrating binding of cancer cell-derived EVs, which expressed TF and PSGL-1 (P-selectin glycoprotein ligand 1), to activated platelets at sites of vascular injury *in vivo*.

Another predictor for cancer-associated thrombosis is an increased number of leukocytes (Hisada et al., 2015). By forming NETs, neutrophils play an important role in thrombus development (Darbousset et al., 2012; von Brühl et al., 2012) and cancer associated thrombosis (Demers et al., 2012; Demers and Wagner, 2014) and it has been shown in a murine breast cancer model that EVs released from tumor cells cooperate with neutrophils in cancer-associated venous thrombosis (Leal et al., 2017). Furthermore, TF+ EVs isolated from breast, ovarian or pancreatic cancer cells can activate platelets thereby aggravating VTE in mouse models (Geddings et al., 2016; Sasano et al., 2021). In human cancer patients, increased levels of circulating TF+ EVs correlate with coagulation activation *in vivo* and increased thrombotic risk (Hron et al., 2007; Haubold et al., 2009). This was supported by observations that cancer patients with VTE presented with increased TF-EV coagulant activity or increased TF+ EVs (Tesselaar et al., 2007; Tesselaar et al., 2009; Zwicker et al., 2009; Manly et al., 2010; Geddings and Mackman, 2013).

**FIGURE 3:**
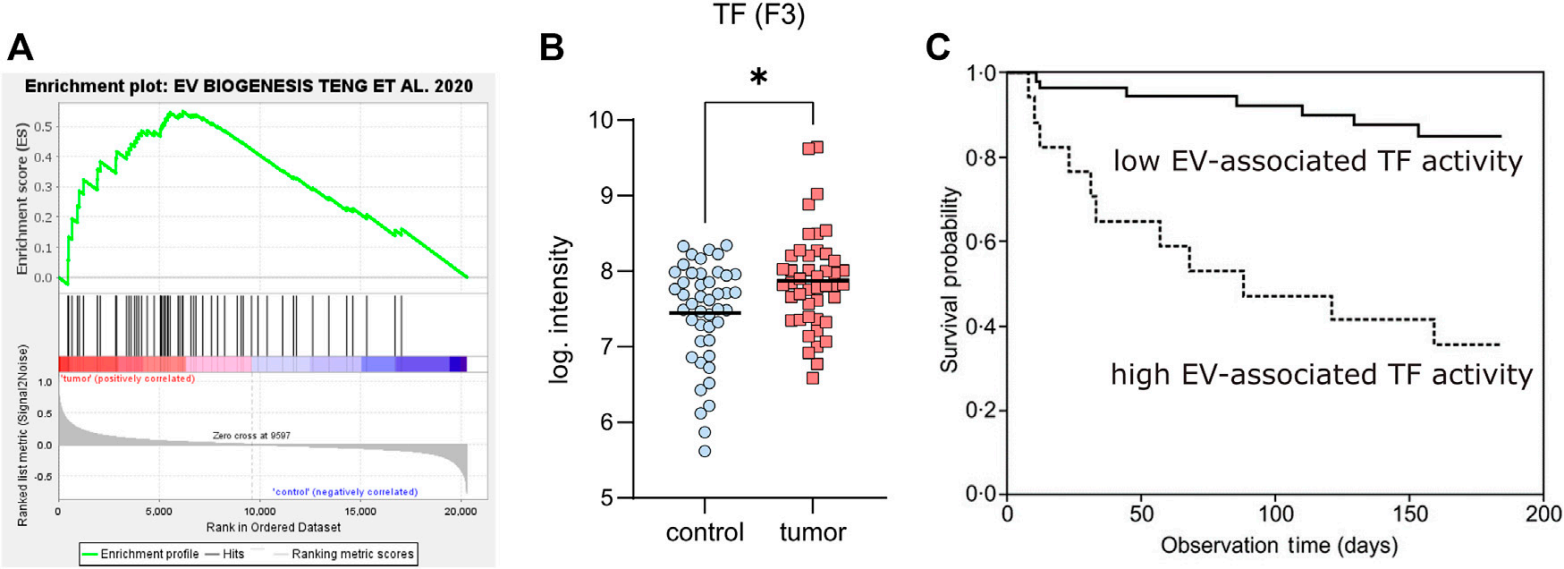
Genes regulating EV-formation, as well as TF are upregulated in cancer. **(A)** Analysis of gene expression changes using micro-array analysis of 45 pancreatic cancer patients (comparing tumor with adjacent non-tumor tissue of the same patients, from (Zhang et al., 2012)). Genes involved in EV formation were deduced from (Teng and Fussenegger, 2021) and used as specific gene set in GSEA (gene set enrichment analysis: free software from the Broad Institute). The plot shows the significant enrichment of EV formation genes (as the enrichment score curve is above the 0-value), implying that these pancreatic cancers release more EVs. **(B)** Tissue factor expression of control and tumor samples from **(A)**. Since the samples do not show a Gauss distribution, a non-parametric test was performed for their comparison (Wilcoxon matched-pairs signed rank test, using GraphPad Prism 9.1), revealing a significant difference between tumors and controls (*p* = 0.0196). **(C)** Kaplan-Meier plot showing the survival probability of patients from the Cancer and Thrombosis Study Vienna (CATS) grouped into patients with low EV-associated TF activity (solid line, below the 75th percentile) and patients with high EV-TF activity (dashed line, above the 75th percentile), published in (Thaler et al., 2013), reproduced here with permission of the publisher.

An important question is certainly, how cancer upregulates both EV formation and TF expression. It has been shown that various oncogenes drive EV formation differently, with some of them upregulating the expression of ESCRT genes, while others such as MYC increase ceramide synthesis. In general, transformed cells tend to downregulate lysosomal degradation pathways and seem to compensate for that by upregulating formation and release of EVs to maintain the membrane homeostasis (Kilinc et al., 2021). To test the hypothesis that cancer cells upregulate genes involved in EV biogenesis we re-analyzed a public dataset of pancreatic ductal adenocarcinoma, which has a high prevalence for thrombotic complications, by gene set enrichment analysis. The dataset contained microarray gene expression values from 45 tumor samples and adjacent non-tumor tissue of the same patients (Zhang et al., 2012). We created a list of all genes, described for EV formation (Teng and Fussenegger, 2021) and uploaded it as local specific gene set (Supplementary Table S1) to the GSEA (gene set enrichment analysis) software (Broad Institute). The following analysis revealed a significant enrichment of this gene set (Figure 3A), supporting the view that malignant transformation can drive EV formation. Next, we analyzed the dataset for TF expression and could verify that the tumor samples express significantly higher levels of this coagulation factor as compared to the adjacent control tissue (Figure 3B).

**FIGURE 4:**
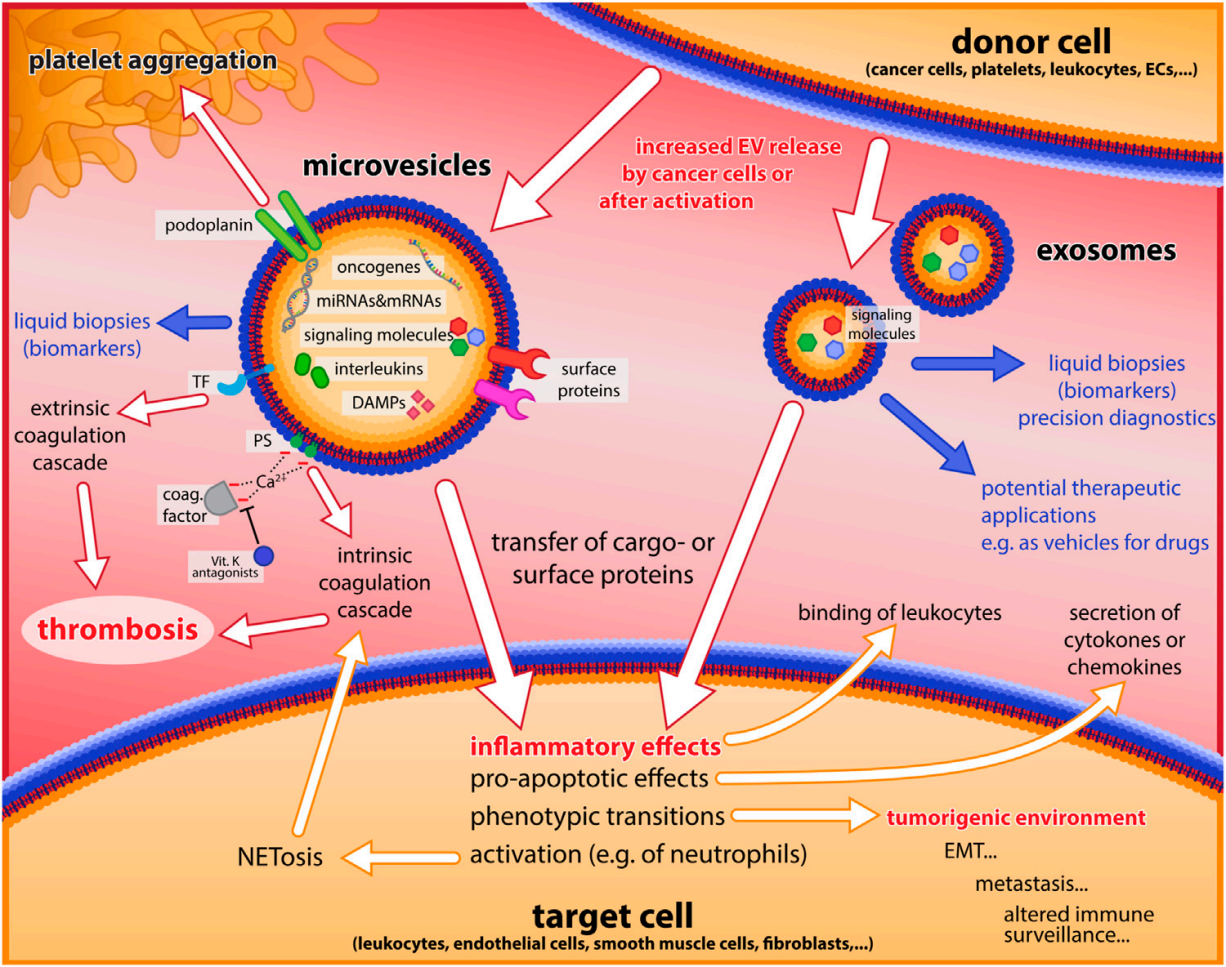
Major roles of EVs in inflammation, cancer, and thrombosis. Microvesicles and exosomes released from donor cells are shown with some of their main constituents, and important effects on inflammatory processes, the coagulation cascade and platelet aggregation, as well as tumorigenic events are indicated by white arrows. Blue arrows and text illustrate potential applications of EVs as biomarkers for diagnostics, and for therapy (as drug vehicles).

These findings are in line with results of the Vienna Cancer and Thrombosis Study (CATS), one of the largest prospective studies to analyze EV-associated TF activity in plasma samples of cancer patients. In a 2-year follow up for the occurrence of symptomatic VTE the study found elevated levels of EV-associated TF activity predominantly in pancreatic and gastric cancer (Thaler et al., 2012). The clinical relevance of these data was confirmed by the observation that patients with a higher EV-associated TF activity had a significantly reduced survival probability (Figure 3C) (Thaler et al., 2013). TF is mostly encrypted and not fully active, when it is exposed in the blood circulation *via* either inflammatory activated ECs, monocytes, or EVs. Therefore, mechanisms that activate TF are important with respect to increased thrombotic risks in cancer. A prime candidate for that is protein disulfide isomerase (PDI), which catalyzes conversion of disulfide bridges known to de-encrypt TF. Indeed, this enzyme has been found upregulated in lung cancer (Ercan et al., 2021) and it might play a role in other cancers as well (Stopa and Zwicker, 2018; Sharda et al., 2021). PDI synergizes with PS on the surface of membranes for activation of TF (Ruf and Langer, 2014), emphasizing the importance of EVs that are positive for TF and expose PS on the outer leaflet of the membrane. Besides EV-associated TF, which primes the plasmatic coagulation cascade, other cancer-related factors may increase the thrombotic risk. One of them is podoplanin, a glycoprotein, which can activate platelets *via* binding to CLEC-2. This protein has a role in embryonic development for the separation of blood and lymphatic vessels (Uhrin et al., 2010) and is expressed by various cancers, where it contributes to thrombotic processes (Riedl et al., 2017; Mir Seyed Nazari et al., 2018). Podoplanin itself increases EV release and has a role in oncogenic transformation (Carrasco-Ramírez et al., 2016). Furthermore, it has been shown that podoplanin-exposing EVs are crucial for thrombotic risks in glioblastoma (Tawil et al., 2021). In summary, it can be stated that both plasmatic coagulation and platelet aggregation can be triggered or supported by EVs, thereby contributing to cancer-associated thrombotic risks. The effects of EVs in inflammation, cancer and thrombosis are summarized in Figure 4.

#### EVs in COVID-19

COVID-19 caused by the SARS-CoV-2 virus (severe acute respiratory syndrome coronavirus type 2) mainly affects the lungs but has also severe effects on multiple other organ systems (cardiovascular, gastrointestinal, hematopoietic, renal, and immune system). The disease is associated with a dysregulated cytokine profile, pathological changes of platelet and lymphocyte numbers, as well as endothelial dysfunction and often involves microthrombosis in the lungs, and acute thrombotic events such as stroke or venous thromboembolism with severe cases presenting with sepsis and multiple organ failure finally leading to death (Fajgenbaum and June, 2020; Koupenova and Freedman, 2020; Elbadawi et al., 2021; Yan et al., 2021). EVs of different cellular origin circulate in plasma of COVID-19 patients (Zaid et al., 2020; Campello et al., 2021) and can trigger distinct metabolic and transcriptional responses in recipient cells (Lam et al., 2021). These EVs contain molecules involved in immune response, inflammation, coagulation and complement pathways (e.g. fibrinogen, fibronectin, complement C1r, serum amyloid P-component, Tenascin) and stimulate respective downstream signaling pathways (Barberis et al., 2021; Lam et al., 2021; Sanwlani and Gangoda, 2021; Sur et al., 2021). Circulating EVs in COVID-19 are often TF and PS-positive and exhibit procoagulant activity indicating that they contribute to disease severity by driving blood clotting (Balbi et al., 2021; Campello et al., 2021; Puhm et al., 2022). Furthermore, it has been suggested that miRNAs contained in EVs contribute to disease progression, e.g. by regulating neutrophil recruitment to the lungs *via* CXCL8 or by binding of TLR4 to the viral spike protein (Meidert et al., 2021). On the other hand, EVs may also contain anti-inflammatory metabolites, which are crucial in coping with overshooting inflammation (Alzahrani et al., 2021).

Interestingly, EVs might also contribute to the spread of the virus infection in the organism as they were also shown to contain viral material (Hassanpour et al., 2020) that they transfer to recipient cells. Based on that, EVs of COVID-19 patients could also be seen as “Trojan horses” that are able to transmit viruses to cells that do not express the ACE2 receptor, thereby contributing to the spread of infection in the host (Wang et al., 2020). This can also induce programmed cell death in recipient cells and trigger further release of EVs (Koupenova et al., 2021; Krishnamachary et al., 2021). A summary of clinical aspects of EV effects is shown in Table 4.

**TABLE 4:** Roles of EVs in clinical conditions.

Disease condition	Effect	EV origin	Molecules	References
Inflammation				
Multiple sclerosis	Pro-inflammatory and anti-inflammatory roles of EVs	Immune cells, mesenchymal stem cells, endothelial cells, glial cells	Fibrinogen, miRNA	Reviewed in (Alberro et al., 2021; Manu et al., 2021)
Rheumatoid arthritis	Procoagulant and pro-inflammatory effects	leukocytes	IL-8, IL-6, RANTES, ICAM-1, VEGF, IgM	Berckmans et al. (2005)
	anti-inflammatory	neutrophils	TGF-β1, annexin 1	(Gasser and Schifferli, 2004; Dalli et al., 2008)
Cardiovascular diseases	diverse	Endothelial cells, blood cells	miRNA	(Sinning et al., 2011; Holnthoner et al., 2017; Desideri et al., 2021)
atherosclerosis	inflammation, vascular dysfunction, leukocyte adhesion and tissue remodeling, coagulation, thrombosis, apoptosis	Platelets, neutrophils	miRNA	(Owens and Mackman, 2011; Chiva-Blanch and Badimon, 2019; Gomez et al., 2020; Hildebrandt et al., 2021)
**Thrombosis**				
	Coagulation	PEV, neutrophils, leukocytes	TF, PS	(Myers et al., 2003; Campello et al., 2012)
**Cancer**				
	Disease progression, coagulation, thrombosis, angiogenesis	Tumor cells, Platelets	DNA, TF, PS, PDI, PDPN	(Tesselaar et al., 2007; Tesselaar et al., 2009; Thaler et al., 2012; Ruf and Langer, 2014; Carrasco-Ramírez et al., 2016; Alberro et al., 2021)
Cancer-associated thrombosis	coagulation		TF, ESCRT genes, Myc, PDPN	(Tesselaar and Osanto, 2007; Tesselaar et al., 2009; Zwicker et al., 2009)
COVID-19	Pro-inflammatory, coagulation and complement pathways, apoptosis	Platelets, endothelial cell	TF, PS, CRP, cytokines, miRNAs, fibrinogen, fibronectin, complement C1r, serum amyloid P-component, Tenascin	(Zaid et al., 2020; Barberis et al., 2021; Campello et al., 2021; Lam et al., 2021; Sanwlani and Gangoda, 2021)
	anti-inflammatory	unknown	Metabolites (e.g.; Lyso-PS)	Alzahrani et al. (2021)

## Discussion

Results of numerous investigations in the last decades demonstrated the importance of EVs for a great variety of processes in biology and (patho-) physiology. While many pioneering studies provided central landmarks for a vivid and fruitful development of the field, the early phase of EV-research apparently suffered from a lack of standardization and suitable controls to justify all conclusions and interpretations that had been made with the available data. This is why clear guidelines as published by the International Society for Extracellular Vesicles (Théry et al., 2018) are so crucial for a further advancement. Sticking to traditions, while being an amiable attitude of human civilization might be a hampering behavior in science – and it is the duty of scientific successors to question the results and interpretations of their predecessors. With that in mind, we propose to examine many of the traditional methods of EV-purification and analysis carefully. We believe that the classical procedure for purifying EVs by differential centrifugation in fixed-angle rotors entails many potential problems, with the difficulty to control whether EVs have been altered or damaged by the process. Therefore, we recommend flotation gradient approaches with swing-out rotors or combinations of techniques such as precipitation, gradient centrifugation and size-exclusion chromatography to prepare reliable material for further research. This should be combined with proper controls for integrity of the vesicles such as digestion experiments with RNases or proteases to confirm that EVs remained intact, and their cargo protected from digestion throughout the isolation and purification procedure. Finally, we always have to consider, whether we are manipulating what we want to investigate, by the used method of material preparation and analysis. Thus, it is also important to consider aspects like the buffer composition or components that are used for the sampling, such as the coagulation inhibitor for blood samples. This has been demonstrated by *ex vivo* increases in EV-numbers in heparin- or citrate-buffers in particular, when samples were agitated (Fendl et al., 2016). Similarly, it is important to keep in mind that sensitive cells such as platelets might be activated *ex vivo*, leading to release of EVs after blood sampling but before analysis.

Besides pitfalls in the isolation and purification of EVs, we also see limitations for analyzing them in a comprehensive manner. The constraints are obvious for flow cytometry techniques, which fail to provide a complete picture of all EVs. While they have the advantage of measuring thousands of particles in a second, they suffer from the inability to detect and measure EVs below 100–150 nm, therefore excluding most of the exosome spectrum. Nanoparticle tracking analysis (NTA) can compensate this in part by detecting much smaller vesicles but has the disadvantage that it cannot differentiate between membranous vesicles and non-membranous particles or protein aggregates such as lipoproteins, which exceed the number of EVs significantly at least in blood samples, and which are also present in fetal calf serum used for cell culture. Applying NTA in fluorescent mode with fluorophores staining membranes or specific EV-components, reduces some of the problems, but cannot provide the richness of data that is delivered by flow cytometry. In many studies, scientists aim for visualizing the structures they are investigating. For that purpose, electron microscopy has always been an important control to demonstrate the membranous nature of EV preparations, but this method is obviously not suited to provide sufficient statistics. An approach that has the potential to fill the gap between cytometry applications, nanoparticle tracking and biochemical analyses is super-resolution microscopy, which can visualize small structures down to about 30 nm resolution and even below. It comes with the option to detect several distinct fluorescence signals and has the potential to deliver good statistics based on automated image analysis (Sigal et al., 2018; Schermelleh et al., 2019; Gwosch et al., 2020). We believe that these methods would allow interesting applications in the field that promote a further advancement.

An aspect that still warrants more attention is the complex mixture of diverse EVs from all different sources and cell types, which are found in biological fluids and in particular the blood circulation. In most studies, EVs from these sources are measured as a bulk entity–and consequently their diversity cannot be reflected sufficiently, even with applications that have a single-particle resolution such as flow cytometry or microscopy. Methods with single-cell readout such as single-cell RNA sequencing, which became popular in other fields, could possibly be applied to EVs in the future. Even though it presents an enormous challenge to obtain a decent number of sequence reads with individual EVs separated by microfluidics, the tremendous development of these techniques lately, might allow an extension to the EV-field. The impressive wealth of novel insights that came along with single-cell methods in the last years in combination with new computational methods like dimensionality reduction approaches for cell clustering would predict a significant advancement of the EV-research field as well – with the potential to discover completely new sets and phenotypes of EVs.

Further, on the preparative side, it might be envisaged that cell-sorting approaches - either by optical means such as fine-tuned Fluorescence Activated Cell Sorting (FACS) down to 100 nm particles or using magnetic beads - might be increasingly applied to isolate different subsets of EVs for further analysis such as transcriptomics or proteomics. Techniques like these may also find application in the diagnostic field, using EVs as valuable biomarker sources and for liquid biopsies.

Finally, we want to emphasize an aspect that is often neglected: the fact that submicroscopic structures such as EVs are so small that quantum effects of components might come into place, meaning that various functionally important components may occur on these structures in a stochastic or binary manner. We would like to illustrate that with a quantitative estimation. If we assume that 3 million proteins are present in one cubic µm of a cell (Milo, 2013), and approximate a cell volume with 1000 μm^3^ (a cube of 10 × 10 × 10 µm), we calculate a number of 3 × 10^9^ proteins per cell. The protein level of TF is estimated for PBMCs or pancreatic cells in the range of 10 ppm (10 parts per million, according to Genecards and ProteomicsDB), which would mean 30,000 TF molecules in a single cell. A cube with 10 µm edge length has a surface of 600 μm^2^. Thus, there will be 50 TF molecules/µm^2^, assuming that all TF is on the cell surface (which is an overestimate, as some TF resides in intracellular membranes such as ER, Golgi and secretory vesicles). If we now compute the surface of various EVs, we will get to about 0.8 µm^2^ for a microvesicle with 0.5 µm diameter and to a surface of 0.008 µm^2^ for an exosome with 50 nm diameter. Accordingly, we calculate a number of 40 TF molecules for the surface of such a microvesicle and a theoretical number of only 0.4 TF molecules for the exosome. These estimations may explain why certain important molecules occur only on a subset of EVs in flow cytometry measurements and they clearly indicate that stochastic “quantum effects” play a role for such small biological entities.

While these considerations are certainly important for our understanding of EV-biology, they do not speak against potential future roles of these structures as vehicles to transfer therapeutics. This translational perspective should certainly be taken seriously – in particular considering the enormous impact that lipid nanoparticles had for vaccines against COVID-19. EVs have the potential to outperform artificial lipid nanoparticles, as they can be functionalized in many ways to target specific cells. Nevertheless, for applications like that, a question has to be answered that is still not clarified in detail: what are the precise mechanisms that lead to fusion of EVs with target cells? Given that cells do not readily fuse with each other, it is still not trivial to understand, how cell-derived EVs with the same membrane topology and composition of the donor cells fuse efficiently with recipient cells *via* a transmembrane fusion process, although some models are already available (Prada and Meldolesi, 2016; Herrmann et al., 2021).

## Glossary

ADP: adenosine diphosphate
aPC: activated protein C
ARDS: Acute respiratory distress syndrome
CD: Cluster of differentiation
COPD: chronic obstructive pulmonary disorder
COVID-19: coronavirus disease 2019
CRP: c reactive protein
DAMP: damage-associated molecular pattern
DC: dendritic cell
EC: endothelial cell
ECM: extracellular matrix
EGFR: epidermal growth factor receptor
EMT: epithelial-mesenchymal transition
ESCRT: endosomal sorting complexes required for transport
EV: extracellular vesicle
FeCl_3_: Iron(III)chloride
GP: glycoprotein
GTP: guanosine triphosphate
HMGB1: high-mobility Group Protein 1
ICAM-1: intercellular adhesion molecule 1
IFN: Interferon
IL: interleukin
IVC: inferior vena cava
LDL: low-density lipoprotein
LFA-1: lymphocyte function-associated antigen 1
MAPK: mitogen-activated protein kinase
MDC: macrophage-derived chemokine
MDSC: myeloid-derived suppressor cell
MHC: major histocompatibility complex
MIP 1a: macrophage inflammatory protein 1a
miR: micro-RNA
MV: microvesicles
MVB: multivesicular body
NET: neutrophil extracellular trap
NF-kB: nuclear factor kappa-light-chain-enhancer of activated B cells
NK: natural killer cell
PAR: protease-activated receptors
PBMC: peripheral blood mononuclear cells
PDI: protein disulfide isomerase
PEV: platelet-derived extracellular vesicles
PS: phosphatidylserine
PSGL-1: P-selectin glycoprotein ligand-1
RBC: red blood cells
SARS-CoV-2: severe acute respiratory syndrome coronavirus 2
SNARE: soluble N-ethylmaleimide-sensitive-factor attachment protein receptor
TF: tissue factor
TLR: toll-like receptor
TNF: tumor necrosis factor
TRAF6: TNF receptor associated factor 6
TRAIL: TNF-related apoptosis-inducing ligand
