# Supplementary material for: Extracellular Vesicles Linking Inflammation, Cancer and Thrombotic Risks
Source: Front Cell Dev Biol. 2022 Mar 17;10:859863. doi: 10.3389/fcell.2022.859863 (PMC8970602; doi:10.3389/fcell.2022.859863)
Supplement: Supplementary file 1 [file Table1.docx]

**Supplementary Table 1**

for:

Extracellular Vesicles Linking Inflammation, Cancer and Thrombotic Risks

Sarah Beck^1,2*^, Bernhard Hochreiter^1^ and Johannes A. Schmid^1*^

^1^Institute of Vascular Biology and Thrombosis Research, Center for Physiology and Pharmacology, Medical Univ. of Vienna, Austria

^2^Institute of Experimental Biomedicine, University Hospital Würzburg, and Rudolf Virchow Center for Integrative and Translational Bioimaging, University of Würzburg, Würzburg, Germany

Gene-Set for EV formation based on the publication of Teng and Fussenegger: 2020:
<https://pubmed.ncbi.nlm.nih.gov/33437589/>

| EV BIOGENESIS Teng et al. 2020 geneset |
| --- |
| doi: 10.1002/advs.202003505 |
| STAM1 |
| STAM2 |
| HGS |
| TSG101 |
| VPS28 |
| VPS37A |
| VPS37B |
| VPS37C |
| VPS37D |
| MVB12A |
| MVB12B |
| UBAP1 |
| EAP30 |
| EAP20 |
| EAP45 |
| CHMP2A |
| CHMP2B |
| CHMP3 |
| CHMP4A |
| CHMP4B |
| CHMP4C |
| CHMP4D |
| CHMP6 |
| CHMP7 |
| CHMP5 |
| CHMP1A |
| CHMP1B |
| VPS4A |
| VPS4B |
| LIP5 |
| PDCD6IP |
| PTPN23 |
| ARF6 |
| SRC |
| PLD2 |
| F2R |
| AP3S1 |
| AP3B1 |
| AP3M1 |
| AP3D1 |
| SMPD2 |
| SMPD1 |
| CD63 |
| RAB31 |
| RAB7 |
| RAB2B |
| RAB9A |
| RAB5A |
| RAB27A |
| RAB27B |
| RAB11 |
| HSPA8 |
| ATG3 |
| ATG7 |
| ATG5 |
| ATG12 |
| MAP1LC3B |
| RALA |
| RALB |
| VAMP7 |
| SYX1A |
| YKT6 |
| SNAP23 |
| PKM |
| VPS4A |
| ARRDC1 |
| ARF1 |
| RHOA |
